# Intraoperative contrast-enhanced urosonography during endoscopic treatment of vesicoureteral reflux in children

**DOI:** 10.1007/s00247-014-2963-7

**Published:** 2014-04-10

**Authors:** Magdalena Maria Woźniak, Paweł Osemlak, Agata Pawelec, Agnieszka Brodzisz, Paweł Nachulewicz, Andrzej Paweł Wieczorek, Maria Małgorzata Zajączkowska

**Affiliations:** 1 Department of Pediatric Radiology, Medical University of Lublin, Al. Racławickie 1, 20-059 Lublin, Poland; 2 Department of Pediatric Surgery and Traumatology, Medical University of Lublin, Al. Racławickie 1, Lublin, Poland; 3 Department of Pediatric Nephrology, Medical University of Lublin, Al. Racławickie 1, Lublin, Poland

**Keywords:** Intraoperative ultrasound, Urosonography, Contrast enhancement, Endoscopic surgery, Vesicoureteral reflux, Child

## Abstract

**Background:**

There are many controversies surrounding the effectiveness of endoscopic treatment of vesicouretheral reflux (VUR) in children, thus it is of highest priority to analyze factors influencing the outcome of therapy and to search for new methods that would increase the success rate and reduce the number of reinjections.

**Objective:**

The aim of the study was to analyze whether intraoperative contrast-enhanced urosonography (ce-US) may increase the effectiveness of endoscopic anti-reflux therapy.

**Materials and methods:**

Intraoperative contrast-enhanced urosonography (ce-US) with SonoVue® was performed in 17 patients (25 ureteral units) undergoing endoscopic treatment of VUR. Ce-US was performed in the operating room before the procedure and after injection of the bulking material. When VUR persisted, the operator repeated the injection, which was followed by ce-US. The results were compared with those obtained from a control group (15 patients; 22 ureteral units).

**Results:**

A repeat injection during a single endoscopic treatment was required in 24% of cases. The overall success rate confirmed at 6–12 months’ follow-ups was 84%. The success rate was significantly higher in comparison to the control group (success: 64%).

**Conclusion:**

Intraoperative ce-US performed during endoscopic treatment of VUR enables immediate monitoring of outcome and provides the opportunity for repeat injection during the same procedure, thus increasing the efficacy of the procedure and reducing the number of reinjections.

**Electronic supplementary material:**

The online version of this article (doi:10.1007/s00247-014-2963-7) contains supplementary material, which is available to authorized users.

## Introduction

Endoscopic injection of bulking agents is one of the methods of treatment of vesicoureteral reflux (VUR) in children, next to long-term antibiotic prophylaxis and open anti-reflux surgery. Owing to a number of benefits such as minimal invasiveness, high efficacy, low complication rate and reduced cost due to short operative time and short hospital stay, it has recently gained great popularity [1]. Following the development and improvement of injection techniques, recent studies have shown higher success rates of endoscopic treatment than open surgery in the treatment of patients with intermediate and high-grade VUR [1, 2]. However, a meta-analysis including 63 studies has demonstrated that the rate of success of endoscopic treatment decreases as the reflux grade increases, from 78.5% in grades I and II down to 51% in grade V [3]. As the volume of the injected material decreases in long-term follow-up, the rate of success also decreases with time from 93% at 1 month after the injection to even 35% after 1 year [4]. There remains controversy regarding the effectiveness of endoscopic treatment, thus it is of high priority to analyze factors influencing the outcome of such therapy and to search for new methods that would increase the success rate, reduce the number of reinjections required and reduce the risk of paravasation or post-interventional ureteric obstruction.

The aim of the current study was to analyze whether intraoperative contrast-enhanced urosonography (ce-US) could be a useful tool for increasing the effectiveness of endoscopic anti-reflux therapy due to immediate monitoring of its outcome during the operation without the risk of radiating imaging such as fluoroscopy and the possibility of repeat injection during a single endoscopic treatment.

## Materials and methods

### Patients

A prospective nonrandomized preliminary study approved by the Research Ethics Committee was performed between December 2011 and October 2013 and informed consent was given by the parents of all participants. Intraoperative ce-US with the off-label use of the US contrast agent SonoVue**®** (Bracco, Milan, Italy) was performed in 17 consecutive patients undergoing endoscopic injection of bulking agents into the submucosa of the 25 ureteral openings in patients treated for vesicoureteral reflux. The mean age of patients was 4 years and 6 months, ranging from 4 months to 15 years and 8 months. The study group consisted of 16 girls and one boy. The inclusion criteria were recurrent urinary tract infections (UTIs) diagnosed on the basis of clinical symptoms and positive urine culture coexisting with vesicoureteral reflux diagnosed with previously performed contrast-enhanced voiding urosonography (ce-VUS). The exclusion criteria involved coexisting abnormalities of the urogenital tract, in particular ureterocele, ectopic ureters, posterior urethral valves, neurogenic bladder due to myelomeningocele and urge incontinence. All patients were monitored for adverse reactions to the contrast agent.

The control group was retrospective and consisted of 15 consecutive patients and 22 ureteral units and included 10 girls and 5 boys undergoing endoscopic injection of bulking agents into the submucosa in a standard protocol, without monitoring by intraoperative ce-US, between July 2009 and December 2011. The mean age of the controls was 3 years and 9 months, ranging from 5 months to 13 years and 2 months. The inclusion criteria were similar as for the study group, e.g., recurrent UTIs diagnosed on the basis of clinical symptoms and positive urine culture coexisting with vesicoureteral reflux diagnosed with previously performed voiding cystourethrography (VCUG). The exclusion criteria involved congenital abnormalities of the urogenital tract, similarly as in the study group. VCUG examinations were performed on an outpatient basis. The contrast agent was administered through the catheter via gravity. Anteroposterior imaging of the bladder was performed during early filling. When bladder filling was complete, steep oblique images centered on the ureterovesical junctions were obtained during voiding. Then the anteroposterior image was performed after voiding was completed.

All patients (*n* = 32) underwent uroflowmetry with post-void residual assessment prior the endoscopic anti-reflux therapy to exclude bladder outlet obstruction as the potential cause of the VUR.

### Procedure

All 32 patients from both the study group (*n* = 17) and control group (*n* = 15) underwent endoscopic dextranomer/hyaluronic acid (Deflux®; Salix Pharmaceuticals, Uppsala, Sweden) injection by subureteric transurethral injection. Endoscopic treatment was carried out as day surgery, with the patients under general anesthesia. Patients received parenteral antibiotic prophylaxis before and after subureteric transurethral injection. The operative technique comprised a subureteric transurethral injection of bulking material with a pediatric cystoscope when the bladder was semi-filled to allow for good visualization of the ureteral orifice and to avoid tension within the submucosal layer of the ureter secondary to overdistension. A 20-gauge needle was inserted 2 to 3 mm proximal to the ureteral orifice and delivered the bulking material underneath the ureter at the 6 o’clock position. The injection proceeded until a “bulge” with an orifice with an elevated, inverted crescent shape was obtained [5]. In both groups, the injected volume varied from 0.5 to 1.3 mL per ureteral unit and was determined according to the age of the patient, shape of the ureteral orifice and the grade of VUR. The volume was recognized as sufficient if the visual assessment performed by the operator was recognized as satisfactory, e.g., if the ureteric orifice was lying on the top of the mountain-shaped bolus. The mean injected volume of bulking agent per renal unit was 0.9 mL in both groups. After the injection, the needle was kept in position for 15–30 s to prevent extrusion of bulking agent. Success of operation was defined as downgrading to grade 0, while failure was defined either as VUR persistence at the same level or decrease of VUR grade. All procedures in both groups were performed by the same operator with 15 years of experience in pediatric urology. The operator was experienced in subureteric transurethral injection, using the same operation technique.

**Fig. 1:**
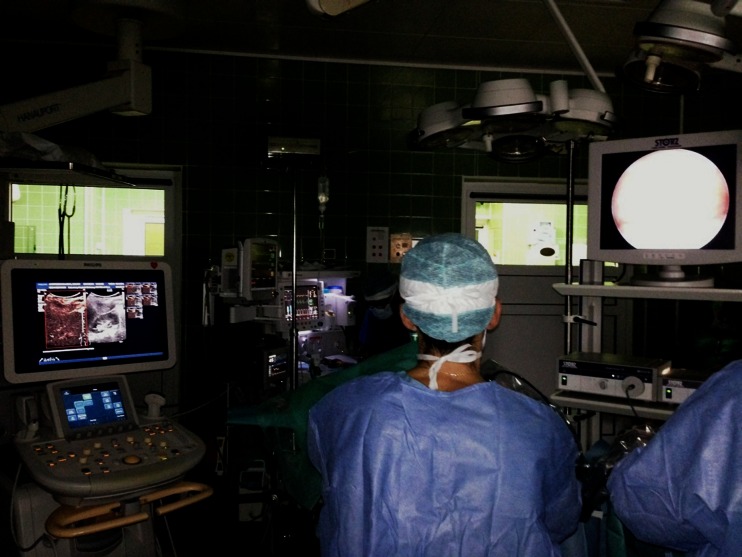
Intraoperative ce-US performed during endoscopic Deflux® injection by subureteric transurethral injection carried out as day surgery, with the patients under general anesthesia

**Fig. 2:**
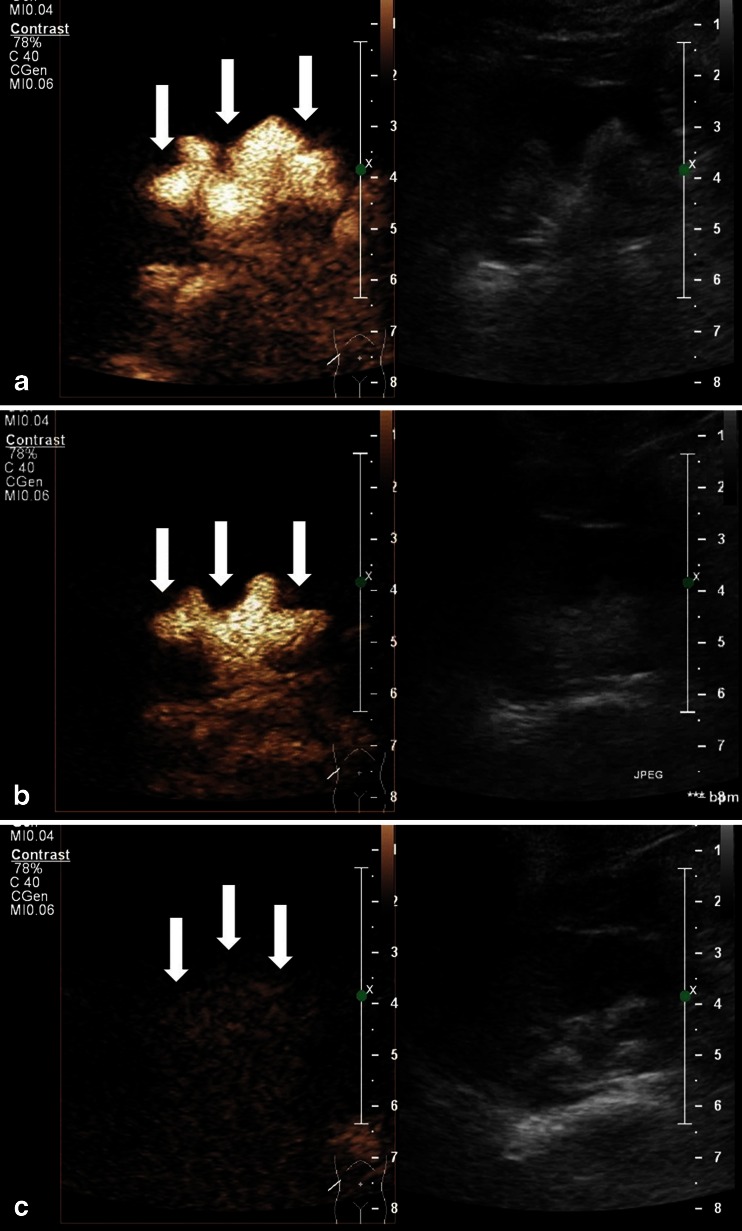
Intraoperative contrast-enhanced urosonography (ce-US). Two-dimensional US in contrast imaging side-to-side mode (contrast image on left side, fundamental mode on right side). **a** Ce-US performed before endoscopic treatment shows right-side low-pressure (passive) VUR grade IV (*arrows*). **b** Ce-US performed after the first injection of bulking material shows downgrading of the right-side VUR to grade III (*arrows*). **c** Ce-US performed after the second injection of bulking material shows no VUR on the right side (*arrows*)

In the patients from the study group (*n* = 17) the procedure, was monitored by intraoperative urosonography (Fig. **1**). Ce-US was performed with the use of a Philips iU22 ultrasound scanner 3–5 MHz transducer using the contrast imaging mode and two-dimensional, side-to-side (grey scale to contrast) technique, according to recommendations for pediatric uroradiology from the Uroradiology Task Force of the European Society of Pediatric Radiology, Pediatric Working Group of the European Society of Urogenital Radiology and results published by other authors [6–8]. At present, ce-US in children can only be performed off-license; however, the authors decided for the off-label use of US contrast agent since, from a medical and scientific perspective, pediatric ce-US should be promoted [6]. A solution of 2.4 ml of the US contrast agent SonoVue® (Bracco, Milan, Italy) and saline (0.9% NaCl) was administered slowly through a catheter (Nelaton, 6–10 F) into the bladder under continuous sonographic monitoring of the bladder and terminal ureters until the maximum bladder capacity was reached while the patient was in the supine position. Subsequently, the catheter was removed and the bladder was emptied. Then the pediatric cystoscope was inserted and the bladder was semi-filled with the solution of saline to perform subureteric transurethral injection. After the bulking agent injection, the bladder was emptied through a cystoscope and the solution of contrast was administered via cystoscope. If the result of the bulking agent injection was not satisfactory the procedure was repeated in the same manner. Overall, ce-US was performed in the operating room while the patient was still anesthetized, either twice or in some patients three times: 1) before the procedure to confirm the presence of VUR, 2) after the injection of the bulking material to assess the efficacy of treatment and 3) after the repeat injection of bulking agent performed in cases when VUR persisted following the initial injection of bulking material (Fig. **2**). The patient was catheterized only for the first ce-US. Subsequent ce-US procedures were performed after the contrast solution administration through the cystoscope. The repeat injection of bulking material was delivered underneath the ureter at the 9 o’clock and/or 3 o-clock position until a “bulge” in the orifice was obtained. Contrast bubbles persisting in the urinary tract from previous ce-US studies were destroyed with the use of the high-MI Power Doppler mode before subsequent fresh administration of contrast solution. All patients were carefully monitored for adverse reactions during all procedures.

All intraoperative ce-US examinations were performed by the same pediatric radiologist with 12 years of experience in pediatric radiology and uroradiology, including ce-VUS.

### Statistical analysis

Statistical analysis was performed using Statistica® (StatSoft, Tulsa, USA) software, version 10.0. The study group and control group were compared in terms of age, sex, occurrence of infections, reflux grade and the volume of the bulging agent injected using the student *t*- and the chi-square tests.

## Results

Low-pressure (“passive”) VUR was diagnosed in patients in the study group (*n* = 17), being unilateral in nine patients and bilateral in eight patients. Among the 25 treated ureteral units, the following grades of VUR were identified: grade II in 3 ureteral units (12%), grade III in 14 ureteral units (56%), grade IV in 6 ureteral units (24%) and grade V in 2 ureteral units (8%). Out of 17 patients, 14 were diagnosed preoperatively based on VCUG and three patients based on ce-VUS. Among pathogens causing the infections the following species of bacteria were found: *Escherichia coli*, *Pseudomonas aeruginosa* and *Proteus* species, at levels ranging from 10^5^/ml to 10^7^/ml.

The control group included 15 children with VUR, which was unilateral in 8 children and bilateral in 7 children. Among 22 treated ureteral units the following grades of VUR were identified: grade II in 5 ureteral units (23%), grade III in 10 ureteral units (45%) and grade IV in 7 ureteral units (32%). All 15 patients were diagnosed preoperatively based on VCUG.

There was no statistically significant difference between the study group and control group in terms of age, occurrence of infections, reflux grade and the volume of the bulging agent injected (*P* > 0.05). There was statistical difference between the groups when sex was compared, as the control group included more boys than the study group (5 vs. 1), which resulted from the fact that the patients were included consecutively.

### Immediate intraoperative results

In the 17 patients undergoing endoscopic injection of bulking agents 25 ureteral units were treated. A single injection of bulking material was sufficient in 19 (76%) ureteral units to obtain success. Success after the second injection performed during the same operation due to persistence of VUR was achieved in three ureteral units (12%). The overall success as assessed with intraoperative ce-US was 88% per ureteral unit. The success rates in grades II, III, IV and V were 100%, 93%, 83% and 50%, respectively. Failure was observed intraoperatively in three (12%) ureteral units (grades III, IV and V), in which even repeat injections of bulking agent did not stop the VUR. In these patients, the ostia were placed laterally, out of the bladder trigon. Altogether, the need for a second injection was observed in six ureteral units (24%) (successful in 3 ureteral units, failed in 3 ureteral units). The results are presented in Table 1. No adverse reactions to US contrast agent were noted.

**Table 1:** The results of endoscopic treatment of vesicouretheral reflux (VUR) obtained with intraoperative contrast-enhanced urosonography in the study group

Study group				Success rate of first injection of bulking material				Success rate of second injection of bulking material				Failure of endoscopic treatment			
Grade	Number of ureteral openings		%	Grade	Number of ureteral openings		%	Grade	Number of ureteral openings		%	Grade	Number of ureteral openings		%
II	3	25	100%	II	3	19	76%	II	0	3	12%	II	0	3	12%
III	14			III	12			III	1			III	1		
IV	6			IV	3			IV	2			IV	1		
V	2			V	1			V	0			V	1		

### Follow-up

Follow-up examinations were performed in all patients 6–12 months after the endoscopic treatment, including the study group and the control group. The children from the study group underwent follow-up ce-VUS, whereas the children from the control group were followed up by VCUG.

In the study group (25 ureteral units), follow-up ce-VUS showed overall success in 21 (84%) ureteral units and failure in 4 (16%). The success rates in grades II, III, IV and V were 100%, 100%, 50% and 50%, respectively. In the group in which treatment was considered to have failed, only high-grade VUR was observed: grade IV in three ureteral units and grade V in one unit.

In the control group (22 ureteral units), follow-up VCUG showed complete success in 14 (64%) ureteral units and failure in 8 (36%) ureteral units (Table **2**). The success rates in grades II, III and IV were 60%, 70% and 57%, respectively.

**Table 2:** The results of long-term endoscopic treatment of vesicouretheral reflux (VUR) obtained at follow-up examinations performed 6–12 months following treatment in the study group and control group

Success				Failure			
Grade	Number of ureteral openings		%	Grade	Number of ureteral openings		%
STUDY GROUP							
II	3	21	84%	II	0	4	16%
III	14			III	0		
IV	3			IV	3		
V	1			V	1		
CONTROL GROUP							
II	3	14	64%	II	2	8	36%
III	7			III	3		
IV	4			IV	3		
V	0			V	0		

## Discussion

The results obtained in this preliminary study show that repeat injection of bulking agent during endoscopic treatment of VUR was needed in 24% of cases when VUR persisted after the first injection. The overall success rate confirmed by follow-up examination performed 6 to 12 months after surgery in the study group was significantly higher than in the control group (84% vs. 63.64%). The overall success rate observed intra-operatively was even higher (88%), with the exception of grade III ureteral units, where intraoperatively a 92.85% success rate was observed, while follow-up showed a 100% success rate. This can be explained either by the bulging agent undergoing a stabilization process with time and sealing the orifice or by natural subsiding of VUR. However, the authors believe that the final effect should be measured in a long-term follow-up up to 5 years following the procedure [9].

With the use of newly available bulking agents it is a reliable and safe alternative to open ureteral reimplantation for the treatment of VUR in children [10]. Since the market for various bulking agents has developed in recent years and the experience of operators has significantly increased over the last decade, endoscopic treatment may become the new gold standard for surgical correction of VUR. However, increasing the effectiveness of endoscopic treatment of children suffering from VUR continues to be a challenge. Various authors have attempted to identify the factors influencing the success rate of endoscopic treatment of VUR. Modifications of the procedure may also play an important role in achieving even better results. The current study belongs to this group, being an attempt to evaluate whether modification of the subureteric transurethral injection technique with implementation of intraoperative monitoring enabling repeated injection of bulking material during the same procedure would increase the success rate and reduce the need for repeat operations/interventions.

Kajbafzadeh et al. [11] attempted to identify independent factors that could predict VUR resolution after endoscopic treatment using dextranomer/hyaluronic acid copolymer (Deflux**®**; Salix Pharmaceuticals, USA) in children free of anatomical anomalies. The study showed that successful VUR correction after the endoscopic injection of Deflux**®** can be predicted with respect to preoperative VUR grade and presence of mound after operation. A similar study was performed by Alkan et al. [12]. The authors attempted to clarify the factors affecting the success rate of endoscopic subureteral injection of VUR, concluding that the procedure provides a high success rate for the treatment of VUR, which decreases to grade V and the presence of bladder exstrophy. A single injection of various materials was found to be successful in most patients with grade II VUR, whereas grade IV and III patients required repeat injections that resulted in 100% and 94% success rates, respectively. These results are consistent with the results obtained herein, where the overall success rate in the study group was 84% and the success rates defined intraoperatively in grades II, III, IV and V were 100%, 92.85%, 83.33% and 50%, respectively; success rates at follow-up were 100%, 100%, 50% and 50% for these grades, respectively.

Elder et al. [3] performed a meta-analysis of the existing literature comparing the results of endoscopic treatment and open surgical correction. The analysis, which included 5,527 patients and 8,101 renal units, showed that the reflux resolution rate (by ureter) following one treatment was 78.5% for grades I and II reflux, 72% for grade III, 63% for grade IV 63% and 51% for grade V. If the first injection was unsuccessful, the second treatment had a success rate of 68%, and the third treatment had a success rate of 34%. The aggregate success rate with one or more injections was 85%, similar to the results obtained herein (84%), with the major difference being that the repeat injection in our study was performed during the same endoscopic treatment. In another study, Elder et al. [3] examined the use of endoscopic injection with dextranomer/hyaluronic acid copolymer as a curative option and as an alternative to antibiotic prophylaxis, concluding that the treatment with endoscopic injection of Dx/HA resulted in significantly fewer urinary tract infections than in children receiving antibiotic prophylaxis [13]. In the study by Garcia-Aparicio et al. [9], the authors demonstrated that in both short- and long-term follow-ups multiple endoscopic treatment of VUR grades II, III and IV was as effective as ureteral reimplantation.

Pichler et al. [14] evaluated whether real-time 3-D-ultrasound together with clinical evaluation could be used as an alternative to VCUG after endoscopic treatment of VUR in children at postoperative follow-up. The authors reported that 3-D-US seems to be sufficient after endoscopic treatment of low-grade VUR, while VCUG should be performed when the depot has shifted, and that invasive investigations are unnecessary in asymptomatic children with orthotopic bulk. In this study all patients underwent follow-up examinations 1 day and 3, 9 and 18 months following treatment. In the modified algorithm suggested in the herein study, owing to the use of intraoperative ce-US during endoscopic treatment, the patients can avoid the follow-up VCUG in the early postoperative period, which seems to benefit the children, aside from all the other advantages of the method. Ce-VUS also appeared to be a reliable diagnostic tool in the follow-up of children undergoing anti-reflux endoscopic treatment [14].

Predicting the outcome of the operation is an important issue, thus Parente et al. [15] evaluated the accuracy of surgeons’ intraoperative observations as a predictor of treatment results. They concluded, however, that the surgeon’s opinion is not an accurate tool to predict the outcome of endoscopic treatment of VUR [15]. Intraoperative ce-US seems to be a promising tool allowing prediction of the final outcome with a higher accuracy than the operator’s opinion by itself.

In the current preliminary study the authors attempted to evaluate whether modification of the standard subureteric transurethral injection endoscopic treatment with bulking agents of VUR, involving the implementation of intraoperative ce-US that enabled the repeated injection of bulking material during the same procedure, would help to increase the success rate. The study group and the control group were comparable in terms of age, occurrence of infections, reflux grade and the volume of the bulging agent injected, and implementation of intraoperative ce-US resulted in a higher success rate in the study group. The preliminary results show that this modification has the potential to increase the success rate and reduce the number of reoperations.

The authors realize that the study has got limitations. A small number of patients in the study group is a major one. Another limitation recognized by the authors is the off-label use of the agent. The authors, however, support the position expressed by the European Society of Paediatric Radiology Uroradiology Task Force and European Society of Uroradiology Paediatric Working Group promoting pediatric ce-US [6]. In herein study no adverse reactions were noted similarly as in other studies performed in pediatric populations. A questionnaire-based European survey, in which data from our center were also included, demonstrated around 4,000 intracavitary (mostly intravesical) pediatric applications of US contrast without any reported major adverse reactions, which was also confirmed in another recent study including 1,010 children [16, 17]. Moreover, performing ce-VUS instead of VCUG in the pediatric population could help significantly reduce the exposure of this group of patients to diagnostic radiation [6, 8, 17–19]. Another important limitation is lack of randomization and the fact that the study group was prospective while it was compared to the control group, which was retrospective. Using a retrospective control group resulted also in the fact that the study group was diagnosed and followed up in ce-VUS while the control group follow-up was in VCUG. It is an important limitation as there is a difference between VCUG and ce-VUS in terms of sensitivity, i.e. on average 10% more VURs are detected with ce-VUS in comparison to VCUG and ce-VUS detects more slightly higher-grade VURs than VCUG [20]. Another important issue is the fact that intraoperative ce-US lacks detection of high-pressure VUR as it would occur only during voiding, which is not achievable in the anaesthetized child. Similarly, the evaluation of the urethra during voiding is not possible, unlike during ce-VUS [20, 21]. Thus, the study should be treated as a preliminary report and requires further studies on a larger population of patients.

## Conclusion

Intraoperative ce-US performed during endoscopic injection of bulking agents enables immediate monitoring of the effect of endoscopic treatment and provides the opportunity for repeat injection of bulking material during a single endoscopic treatment, increasing the efficacy of the procedure and reducing the number of re-injections required. In comparison to the control group the results obtained show that the method enables a significantly higher success rate. Additionally, it enables a more precise prediction of the final outcome of the operation while still in the operating room. However, the long-term clinical results of endoscopic treatment should be evaluated with a follow-up of at least 5 years after the operation. Further studies on larger populations are mandatory.

## Electronic supplementary material

Below is the link to the electronic supplementary material.

ESM 1 (AVI 2553 kb)
